# Maternal Transfer of Natural (Auto-) Antibodies in Chickens

**DOI:** 10.3382/ps/pez017

**Published:** 2019-01-25

**Authors:** M Rifqi Ismiraj, Joop A J Arts, Henk K Parmentier

**Affiliations:** Section of Immunology, Adaptation Physiology Group, Department of Animal Sciences, Wageningen University, De Elst 1, 6708 WD Wageningen, the Netherlands

**Keywords:** natural (auto-) antibodies, poultry, divergent selection, maternal transfer

## Abstract

The presence and relative levels (titers) of IgM and IgG natural antibodies (**NAb**) binding keyhole limpet hemocyanin (**KLH**), and natural (auto-) antibodies (**N(A)Ab**) binding salmon double-stranded DNA (**dsDNA**), (oxidated-) phosphatidyl (phosphoryl) choline-conjugated bovine serum albumin (**PC-BSA**), PC-conjugated ovalbumin (**PC-OVA**), and OVA, respectively, were studied in adult hen plasma, egg yolk, egg albumen, plasma of their hatchlings, and in 8-day-old chick plasma. Birds and eggs were from 2 lines divergently selected for high or low **NAb** levels binding KLH. This study aimed to determine 1) correlated phenotypic responses of selection for NAb to KLH, 2) transfer of maternal NAb and N(A)Ab via egg compartments, 3) levels of likely maternal NAb and N(A)Ab in hatchlings and 8-day-old chicks, and 4) whether a composite trait: IgM anti-PC-BSA/IgG anti-dsDNA ratio in the compartments could be used as a parameter for health or immune status.

NAb and N(A)Ab to all tested antigens were found in adult hens, but low or no levels were found for IgM in yolk and IgG in albumen. Depending on the antigen, NAb and N(A)Ab were found in hatchlings and day 8 birds. Divergent selection and breeding based on NAb binding KLH affected antibody titers of almost all antigens in almost all compartments, in a similar way. Maternal transfer of NAb and N(A)Ab from the adult hen to offspring was via specific routes for specific antigens and isotypes, especially for IgG as suggested by cluster analyses and significant correlations. There was little indication of production of new NAb and N(A)Ab to the studied antigens in either the egg compartments or the hatchlings. A composite trait of IgM PC-BSA/IgG dsDNA ratio was as yet not indicative for immune status, as no significant differences were found between the lines for all compartments.

In conclusion, hens provide neonatal chickens with natural (self-) binding IgG antibodies that have been proposed to perform homeostatic functions during the period in which neonates do not produce these antibodies themselves.

## INTRODUCTION

Natural antibodies (NAb) and natural auto-antibodies (N(A)Ab) are antibodies present in normal healthy individuals, without any known previous exposure to these antigens (Avrameas, 1991; Xu et al., 2015). NAb are regarded as important specific components of the first line of (innate) defense in cooperation with for instance the complement system against invading antigens or microbes. N(A)Ab likely perform a homeostatic role in clearing ‘waste’ such as intracellular molecules or modified self (cell or nuclear surface structures of necrotic cells, and tumor cells), especially ‘danger’ related antigens (Lutz et al., 2009; Schwartz-Albiez et al., 2009) such as oxidated phosphatidyl choline: phosphoryl choline- (PC) conjugated molecules (Binder, 2012), or dsDNA (Lòpez et al., 2016) thereby preventing harmful autoimmune and inflammatory reactions. In mice, CD5^+^ (B1) B cells are the principal source of NAb and N(A)Ab (Herzenberg, 2000; Ansel et al., 2002; Hardy & Hayakawa, 2005). These B1 B cells differ from conventional B2 B cells in showing innate-like instead of adaptive-like characteristics (Baumgarth, 2011). To our knowledge, B1 cells have not been identified in poultry. Both NAb and N(A)Ab have a broad specificity repertoire with highly polyspecific (cross-reactive) receptors and usually a low binding affinity (Casali and Notkins, 1989; Trenynck and Avrameas, 1986), which can be directed to foreign (microbial) as well as self-antigens (Avrameas et al., 1981, Boes, 2000; Boes et al., 2000; Baumgarth, 2011).

In this study, we selected 5 antigens that may represent self (OVA), ‘danger’ PC-conjugated self (PC-OVA), non-self (PC-BSA), dsDNA, and foreign non-danger (KLH) to chickens. PC has been indicated to ‘endanger’ antigens (Binder, 2012). Double-stranded DNA (dsDNA) represents a ‘self-antigen’ often associated with nuclear disruptions especially in necrotic cells that is released into the extracellular milieu. Elevated levels of IgG anti-dsDNA antibodies may reflect inflammation and were shown to downregulate the production of IgM anti-PC antibodies, suggesting that a composite trait between those two antibodies may be informative for health status (López et al., 2016). Keyhole limpet hemocyanin (KLH) is regularly used as an example of a naive antigen for detecting NAb in laying hens. High levels of NAb binding KLH were associated with enhanced survival of layers during a laying period (Star et al., 2007; Sun et al., 2011, 2013).

The present study addressed the presence and levels of NAb and N(A)Ab in plasma of adult hens from 2 White Leghorn lines divergently selected and bred for high (**H line**) or low (**L line**) titers of NAb binding KLH during 4 generations, in yolk and albumen of their eggs, in plasma obtained at hatch (hatchlings), and finally in 8–day-old chicks against PC-BSA, KLH, dsDNA, PC-OVA, and OVA, respectively. Two antibody isotypes (IgM and IgG) were measured using ELISA. Correlation and cluster analysis were done to reveal maternal antibody transfer between adult hens and their offspring via the yolk and albumen to the hatchling. Finally, the ratio between IgM PC-BSA and IgG dsDNA titers was estimated to assess whether such a composite trait that has been proposed as a parameter for health is also informative for immune status, since the two lines differ in the levels of NAb binding KLH.

## MATERIAL AND METHODS

### Birds

Birds originated from the Hendrix Genetics pure bred elite WA White Leghorn layer line.

### Antibody Titers in Plasma and Egg Compartments

Plasma samples were from hens from 3 different ages: adult hens (35 wk of age, 15 birds from 2 lines: High (H) and Low (L) lines divergently selected for 4 generations for high or low levels of total antibodies binding KLH at 16 wk of age (Berghof et al., 2018), their hatchlings (15 chicks, 1 per hen from both lines), and 8-day-old female chicks (15 birds from both lines), which were not progeny of the hens. Plasma from hatchlings was gathered immediately at hatch. The eggs used in this study (15 from both lines) were from the adult hens. Yolk and albumin were separated using chloroform extraction as described by Hagan et al. (2004) with minor modifications.

Titers of IgM and IgG antibodies binding the various antigens were determined in individual plasma or egg compartment samples by an indirect ELISA as follows: 96-well flat-bottomed medium binding ELISA plates (Greiner Bio-One, Alphen aan de Rijn, the Netherlands) were coated with 100 μL of coating buffer (pH 9.6) containing antigens with specific concentrations (see Appendix 1) and incubated at 4°C overnight. The antigens used were PC-BSA (LGC Biosearch Technologies, Petaluma, CA, USA.), KLH (MP Biomedicals Inc., Aurora, OH, USA), Salmon dsDNA (Sigma-Aldrich, Darmstadt, Germany), PC-OVA (ChemCruz, Dallas, TX, USA), and OVA (Sigma-Aldrich). A standard positive plasma sample was stepwise diluted in duplicate (columns 11 and 12 on each plate). After subsequent washing, the plates were filled with 100 μL of PBS containing 0.05% Tween 20 and 1% horse serum per well. Plasma samples were stepwise 2- or 4-fold diluted starting at 1:10 or 1:40 dilution (Appendix 1), and the plates were incubated for 1 h at room temperature. After washing, plates were incubated with specific conjugates and specific optimized dilutions (Appendix 1) and incubated for 1.5 h at room temperature. The conjugates used were goat polyclonal anti-chicken IgM and goat polyclonal anti-chicken IgG-Fc both conjugated to horse radish peroxidase (**PO**) (Bethyl Laboratories Inc., Montgomery, TX, USA). After washing, binding of the antibody isotypes in the plasma and egg samples to the antigens was visualized by adding 100 μL of substrate (70 μg/mL tetramethylbenzidine and 0.05% H_2_O_2_). After 10 min, the reaction was stopped with 50 μL of 1.25 M H_2_SO_4_. Extinctions were measured with a Multiskan GO (Thermo Fisher Scientific, Breda, the Netherlands) at a wavelength of 450 nm. Titers were calculated based on log_2_ values of the dilutions that gave an extinction closest to 50% of EMAX, where EMAX represents the mean of the highest extinction of the standard positive plasma sample (columns 11 and 12) present on each plate. Titers below 1 were regarded as being absent or not detectable and therefore not included in the statistical analysis.

### Composite Trait

A composite trait was calculated based on the ratio of IgM anti-PC-BSA titer/IgG anti-dsDNA titer (López et al., 2016) in each compartment (hens, egg yolk, hatchlings, and day 8 chicks) except for egg albumin where IgG anti-dsDNA was lacking, and compared between the High and Low line.

### Statistical Analysis

To detect significant line differences, a one-way ANOVA for line was performed for the various compartments. To determine maternal antibody transfer, a partial correlation for compartments adjusted for line was performed. Analyses were done with SAS statistics 9.2 (proc GLM for ANOVA and proc CORR for correlation) (SAS Institute Inc., 2008). The level of significance used was *P* < 0.05. Clustering of lines and individuals based on antibody titers against antigens of the 2 isotypes was done by principal component analysis (**PCA**) followed by redundancy analysis (**RDA**) using Monte Carlo unrestricted full permutation test in the CANOCA 4.5 package for Windows (Lepš & Šmilauer, 2003).

## RESULTS

### Presence and Levels of Antibodies in Various Compartments and the Effect of Line

#### Presence of Antibodies and Line Effect on Antibody Titers in Adult Hens.

As shown in Table 1, antibodies from both isotypes and binding all antigens were present in plasma of 35-wk-old hens. In the H-line hens, titers were all significantly higher than in the L-line hens. The mean titer differences ranged 1–2 units (i.e., 2- to 4-fold level differences) between H-line and L-line birds for all antigens and isotypes.

**Table 1.** The effect of line on IgM and IgG titers of plasma samples collected from high (H) line and low (L) line hens at 35 wk of age (n = 15 hens per line).

Antigen	Line	LSMeans	SE	*P*-value	Remarks
PC-BSA IgM	H	9.15	0.22	***	H > L
	L	7.11			
PC-BSA IgG	H	7.95	0.39	**	H > L
	L	6.27			
KLH IgM	H	8.47	0.25	***	H > L
	L	6.26			
KLH IgG	H	7.59	0.48	***	H > L
	L	5.08			
dsDNA IgM	H	9.15	0.41	***	H > L
	L	6.86			
dsDNA IgG	H	8.78	0.51	**	H > L
	L	6.77			
PC-OVA IgM	H	7.31	0.25	***	H > L
	L	5.53			
PC-OVA IgG	H	6.87	0.45	*	H > L
	L	5.35			
OVA IgM	H	8.80	0.28	***	H > L
	L	7.05			
OVA IgG	H	6.33	0.40	**	H > L
	L	4.43			

*P < 0.05, **P < 0.01, ***P < 0.001

#### Presence of Antibodies and Line Effect on Antibody Titers in Yolk.

In general (see Table 2), some IgM antibodies were not detected (PC-OVA, dsDNA) or were very low, such as IgM binding KLH in the L line, dsDNA, or OVA, respectively. When IgM antibodies were found in the yolk, there were no significant differences between H- and L-line samples for IgM antibodies binding PC-BSA and OVA. IgG antibodies to all antigens were found in the yolk, and significant line differences in IgG antibody titers for PC-BSA and KLH were found: H-line samples having higher titers than L-line samples. IgG titers to dsDNA, PC-OVA, and OVA were not affected by line. High IgG titers binding OVA were found in yolk of both lines.

**Table 2.** The effect of line on IgM and IgG antibody titers in high (H) line and low (L) yolk (n = 15 per line).

Antigen	Line	LSMeans	SE	*P*-value	Remarks
PC-BSA IgM	H	5.01	0.69	NS	H = L
	L	3.34			
PC-BSA IgG	H	5.25	0.48	*	H > L
	L	3.81			
KLH IgM	H	NF	–	–	
	L	2.08	0.49		
KLH IgG	H	4.32	0.23	***	H > L
	L	3.11			
dsDNA IgM	H	NF	–	–	
	L	NF	–	–	
dsDNA IgG	H	5.27	0.34	NS	
	L	4.33			
PC-OVA IgM	H	NF	–	–	
	L	NF	–	–	
PC-OVA IgG	H	4.85	0.23	NS	
	L	4.41			
OVA IgM	H	1.03	0.20	NS	
	L	1.35			
OVA IgG	H	9.50	0.25	NS	
	L	9.11			

NF: Not Found.

NS: not significant, *P < 0.05, ***P < 0.001

#### Presence of Antibodies and Line Effect on Antibody Titers in Albumen.

IgM antibodies binding all antigens were found in the albumen, especially in the H line, whereas IgG antibodies were low or absent (Table 3). Only IgG binding OVA was found in the albumen of both lines. Titers of IgM antibodies binding PC-BSA, KLH, dsDNA, and PC-OVA, respectively, were very low or absent in the L-line albumen.

**Table 3.** The effect of line on IgM and IgG antibody titers in High (H) line and Low (L) line albumen (n = 15 per line).

Antigen	Line	LSMeans	SE	*P*-value	Remarks
PC-BSA IgM	H	2.20	0.31	–	
	L	NF	–		
PC-BSA IgG	H	NF	–	–	
	L	NF	–	–	
KLH IgM	H	4.05	0.33	***	H > L
	L	1.50	0.36		
KLH IgG	H	NF	–	–	
	L	NF	–	–	
dsDNA IgM	H	1.20	0.17	–	
	L	NF	–		
dsDNA IgG	H	NF	–	–	
	L	NF	–		
PC-OVA IgM	H	2.96	0.34	**	H > L
	L	0.91	0.36		
PC-OVA IgG	H	NF	–	–	
	L	NF	–	–	
OVA IgM	H	2.63	0.74	NS	
	L	1.71	0.79		
OVA IgG	H	2.46	1.63	NS	
	L	2.32	1.26		

NF: not found.

NS: not significant, **P < 0.01, ***P < 0.001

#### Presence of Antibodies and Line Effect on Antibody Titers in Hatchlings.

Both isotypes binding all antigens were found in plasma at hatch (Table 4) but not IgM antibodies binding KLH. Significantly higher titers were found in the H-line hatchlings for IgM antibodies binding dsDNA and PC-OVA, respectively, and IgG antibodies binding KLH, dsDNA, PC-OVA, and OVA, respectively.

**Table 4.** The effect of line on IgM and IgG antibody titers in plasma at hatch from high (H) line and low (L) line chicks (n = 15 chicks per line).

Antigen	Line	LSMeans	SE	*P*-value	Remarks
PC-BSA IgM	H	3.05	0.22	NS	
	L	2.63			
PC-BSA IgG	H	6.45	0.37	NS	
	L	5.43			
KLH IgM	H	NF	–	–	
	L	NF	–	–	
KLH IgG	H	6.38	0.43	**	H > L
	L	4.65			
dsDNA IgM	H	2.79	0.21	***	H > L
	L	1.11			
dsDNA IgG	H	7.57	0.41	**	H > L
	L	5.65			
PC-OVA IgM	H	NF	–	–	
	L	NF	–	–	
PC-OVA IgG	H	5.67	0.41	*	H > L
	L	4.47			
OVA IgM	H	1.05	0.25	NS	
	L	1.18			
OVA IgG	H	5.28	0.28	***	H > L
	L	3.43			

NF: not found.

NS: not significant, *P < 0.05, **P < 0.01, ***P < 0.001

#### Presence of Antibodies and Line Effect on Antibody Titers at Day 8 of Age.

Both isotypes binding all antigens were found in plasma at day 8 of age in the H-line birds (Table 5). IgM and IgG antibodies binding PC-BSA, KLH, dsDNA, PC-OVA, and OVA were absent in the L line day 8 birds. IgM antibodies binding dsDNA, PC-OVA, and OVA were absent in both lines. On the whole, titers of both isotypes, when present, binding all antigens were higher in the H-line plasma samples.

**Table 5.** The effect of line on IgM and IgG antibody titers in plasma from high (H) line and low (L) line chicks at 8 days of age (n = 15 chicks per line).

Antigen	Line	LSMeans	SE	*P*-value	Remarks
PC-BSA IgM	H	1.47	0.19	–	
	L	NF	–		
PC-BSA IgG	H	2.12	–	–	
	L	NF			
KLH IgM	H	NF	–	–	
	L	NF			
KLH IgG	H	4.89	0.26	***	H > L
	L	2.50			
dsDNA IgM	H	NF	–	–	
	L	NF			
dsDNA IgG	H	4.95	0.22	***	H > L
	L	3.35			
PC-OVA IgM	H	NF	–	–	
	L	NF	–		
PC-OVA IgG	H	3.92	0.30	0.0017	H > L
	L	2.43			
OVA IgM	H	NF	–	–	
	L	NF1	–		
OVA IgG	H	2.99	0.22	***	H > L
	L	1.51			

NF: not found

***P < 0.001

#### Correlations between Hen, Egg Compartments, and Hatchling

##### Correlation via the Yolk Route.

Table 6 shows the correlation values of antibody titers between adult hen plasma and yolk, and between yolk and hatchlings corrected for line. Significant positive correlations between hen plasma and yolk were found for IgG binding PC-BSA in both lines, IgG antibodies binding KLH, dsDNA, and OVA in the H line, and PC-OVA in the L line. Significant positive correlations between yolk and hatchlings were found for IgG antibodies binding PC-BSA and PC-OVA, respectively, and IgM antibodies binding dsDNA in both lines. In the H line, significant positive correlations were found for IgM antibodies binding PC-BSA, and IgG antibodies binding KLH, and dsDNA, respectively.

**Table 6.** Correlation between IgM and IgG antibody titers in hen-yolk-hatchling samples.

	Description Pearson's	Hen-Yolk		Yolk-Hatchlings	
Antigen	R *P*-value	High Line	Low Line	High Line	Low Line
PC-BSA IgM	R	0.20	0.41	0.79	−0.05
	P	NS	NS	**	NS
PC-BSA IgG	R	0.85	0.76	0.60	0.68
	P	***	***	*	**
KLH IgM	R	–	0.04	–	–
	P	–	NS	–	–
KLH IgG	R	0.90	0.46	0.87	0.01
	P	***	NS	***	NS
dsDNA IgM	R	–	–	–	–
	P	–	–	–	–
dsDNA IgG	R	0.84	0.14	0.80	0.29
	P	***	NS	**	NS
PC-OVA IgM	R	–	–	–	–
	P	–	–	–	–
PC-OVA IgG	R	−0.25	0.59	0.68	0.52
	P	NS	*	**	*
OVA IgM	R	−0.10	0.14	0.14	0.16
	P	NS	NS	NS	NS
OVA IgG	R	0.74	0.09	0.48	0.26
	P	**	NS	NS	NS

–Not calculated.

NS: not significant, *P < 0.05, **P < 0.01, ***P < 0.001

##### Correlation via the Albumen Route.

The correlations between adult hen plasma and egg albumen, and albumen and hatchling corrected for line are shown in Table 7. Correlations were only found for IgM antibodies binding PC-BSA, dsDNA, and OVA in the H line and OVA in the L line, but not significant. Also not-significant correlations were found for IgG binding OVA in both lines.

**Table 7.** Correlation between IgM and IgG antibody titers in hen-albumen-hatchling samples.

	Description Pearson's	Hen-Albumen		Albumen-Hatchlings	
Antigen	R *P*-value	High Line	Low Line	High Line	Low Line
PC-BSA IgM	R	0.33	–	−0.12	–
	P	NS	–	NS	–
PC-BSA IgG	R	–	–	–	–
	P	–	–	–	–
KLH IgM	R	0.38	0.57	–	–
	P	NS	*	–	–
KLH IgG	R	–	–	–	–
	P	–	–	–	–
dsDNA IgM	R	0.45	–	0.24	–
	P	NS	–	NS	–
dsDNA IgG	R	–	–	–	–
	P	–	–	–	–
PC-OVA IgM	R	0.32	–	–	–
	P	NS	–	–	–
PC-OVA IgG	R	–	–	–	–
	P	–	–	–	–
OVA IgM	R	0.74	0.49	0.46	0.42
	P	**	NS	NS	NS
OVA IgG	R	−0.09	−0.11	0.25	0.03
	P	NS	NS	NS	NS

–Not calculated

NS: not significant, *P < 0.05, **P < 0.01

##### Correlation of Adult Hen and Hatchling.

Table 8 shows the correlations between adult hen plasma and plasma at hatch corrected for line. Significant positive correlations between adult hen plasma and plasma at hatch were found for IgG antibodies binding PC-BSA, KLH, dsDNA, PC-OVA, and OVA, respectively, for both lines, and IgM antibodies binding dsDNA, and OVA, respectively, only in the H line.

**Table 8.** Correlation between IgM and IgG antibody titers in hen and hatchling plasma samples.

	Description Pearson's		
Antigen	R *P*-value	H Line	L Line
PC-BSA IgM	R	0.23	−0.01
	P	NS	NS
PC-BSA IgG	R	0.79	0.87
	P	***	***
KLH IgM	R	–	–
	P	–	–
KLH IgG	R	0.93	0.68
	P	***	**
dsDNA IgM	R	0.72	0.07
	P	**	NS
dsDNA IgG	R	0.91	0.76
	P	***	**
PC-OVA IgM	R	–	–
	P	–	–
PC-OVA IgG	R	0.87	0.76
	P	***	***
OVA IgM	R	0.52	−0.26
	P	*	NS
OVA IgG	R	0.80	0.55
	P	***	*

–Not calculated

NS: not significant, *P < 0.05, **P < 0.01, ***P < 0.001

##### Clustering by Principal Component Analysis.

**Figure 1.**
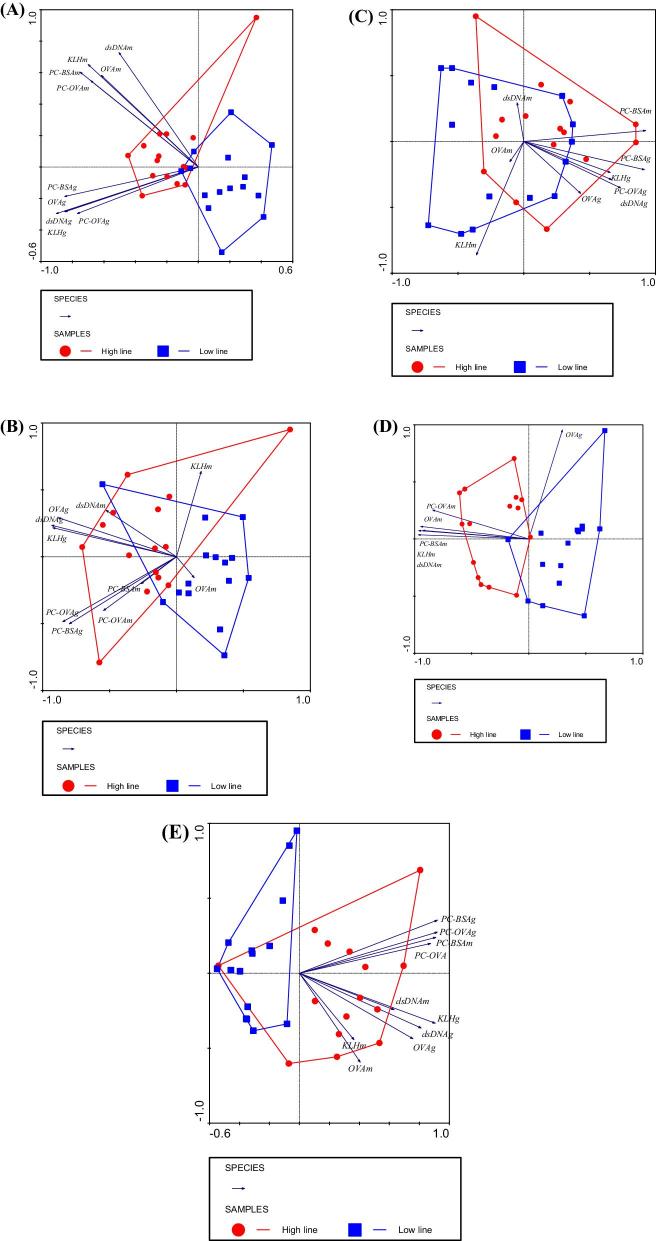
Grouping by PCA of adult hens (A), hatchlings (B), yolk (C), albumen (D), and day 8 chicks (E) on the basis of IgM and IgG antibody titers to 5 antigens: KLH, PC-BSA, PC-OVA, dsDNA, and OVA. Diagrams include 15 birds or specimens from each line (enveloped) that were analyzed with ELISA. ‘Eigen values’ explaining the amount of variation were 60% horizontal and 20% vertical (A); 64% horizontal and 13% vertical (B); 58% horizontal and 17% vertical (C); 74% horizontal and 19% vertical (D); 73% horizontal and 12% vertical (E). Diagrams include antigen and isotype combinations (arrows). Length and direction of arrows indicate the contribution of the antigen–isotype combination to the grouping of the lines. Small fonts after the antigen label represent the isotype m: IgM and g: IgG.

Figure 1 shows the clustering of the H and L lines in different compartments based on binding activity against specific antigens by the two isotypes. Data points (individual hens) are enveloped by line. In adult hens, there is distinct grouping of each line (Figure 1A) with little overlap. All the antibody–antigen combinations (arrows) contributed to the distinct grouping of the two lines. Redundancy analysis (RDA) revealed that the H line hens were mostly characterized by IgM to PC-BSA (arrow PC-BSAm) and IgG to KLH (arrow KLHg) in Figure 1A, respectively. Also for the hatchlings, a grouping between lines is visible (Figure 1B), but there is more overlap illustrated by the envelopes. As in the adult hens, the antibody–antigen combinations contributed to the grouping of the lines apart from IgM binding KLH (arrow KLHm) and OVA (arrow OVAm). The H-line hatchlings are mostly characterized by IgM to dsDNA (arrow dsDNAm) and IgG to OVA (arrow OVAg) (Figure 1B), respectively. For egg yolk (Figure 1C), the most informative (RDA) isotype for clustering is IgG to KLH (arrow KHLg) and OVA (arrow OVAg), respectively. Figure 1C also shows that most antibody–antigen combinations contribute to the grouping of the lines apart from IgM binding dsDNA (arrow dsDNAm), OVA (arrow OVAm), and KLH (arrow KLHm), respectively. Overlap between the 2 lines is indicated by the envelopes, and grouping is less distinct compared to adult hen (Figure 1A) or hatchling (Figure 1B). For egg albumen (Figure 1D), RDA revealed that the most informative isotype is IgM binding dsDNA (arrow dsDNAm) and PC-OVA (arrow PC-OVAm), respectively. In egg albumen, apart from IgG binding OVA (arrow OVAg) the IgM antibodies binding PC-OVA (arrow PC-OVAm), OVA (arrow OVAm), PC-BSA (arrow PC-BSAm), KLH (arrow KLHm), and dsDNA (arrow dsDNAm) contributed to the clearly visible grouping of the lines. In day 8 chicks (Figure 1E), all antibody–antigen combinations contributed to the grouping of the lines. Grouping of day 8 chicks is visible, and alike adult hens (Figure 1A). Overlap is caused by 1 individual low-line bird. RDA revealed that IgG to KLH (arrow KLHg) is the most characteristic antibody of the H line day 8 chicks alike in adult hens.

**Figure 2.**
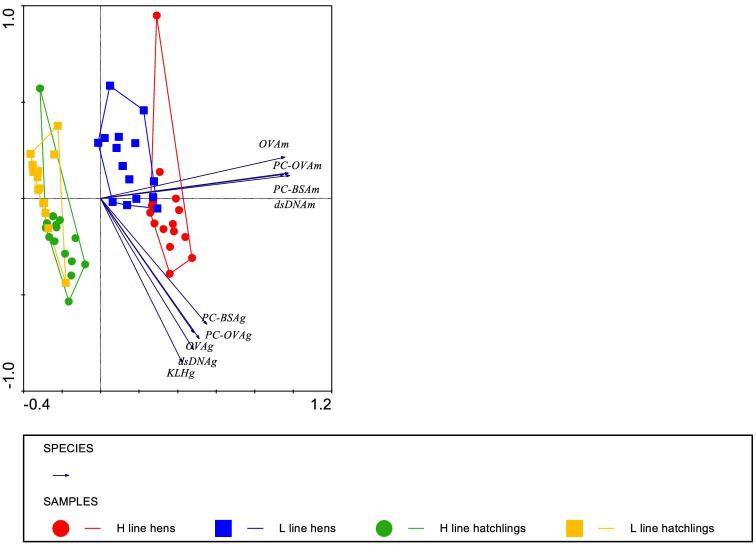
Grouping of adult hens and their hatchlings on the basis of IgM and IgG antibody titers to 5 antigens: KLH, PC-BSA, PC-OVA, dsDNA, and OVA. Ordination plots by principal component analysis. Diagrams include 14 to 15 birds (hens and hatchlings) from each line (enveloped) that were analyzed with ELISA. ‘Eigen values’ explaining the amount of variation were 73% horizontal and 18% vertical. Diagram includes antigen and isotype combinations. Length and direction of arrows indicate the contribution of the antigen-isotype combination to the grouping of the lines. Small fonts after the antigen label represent the isotype m: IgM and g: IgG.

Figure  2 shows the grouping by PCA of H- and L-line hens and their hatchlings. All antibody–antigen combinations contributed to the grouping of the 2 lines and the 2 ages, but the angle of almost 45^o^ between the IgM antigen combinations and the IgG antigen combinations suggested independent contributions. Adult hens show distinct grouping, whereas in this PCA, hatchlings of both lines are more alike suggesting that the line differences develop during ageing from hatch to 16 wk of age: the selection criterion. Hatchlings lacked IgM antibodies to KLH in plasma, which therefore could not be used in the analysis.

##### Composite Trait of the IgM Anti-PC-BSA and IgG Anti-dsDNA Ratio.

Table 9 shows that the comparison of composite traits revealed no significant differences between the lines in the various compartments and ages.

**Table 9.** Comparison of IgM anti-PC-BSA/IgG anti-dsDNA ratios (composite trait) in high (H) line and low (L) line compartments.

Compartment	Line	LSMean	SE	*P*-value
Hen	H	1.16	0.12	0.81
	L	1.12		
Yolk	H	1.06	0.18	0.16
	L	0.70		
Hatchlings	H	0.42	0.05	0.28
	L	0.50		
Day 8	H	0.29	0.04	
	L	–	–	

–Not calculated

## DISCUSSION

Two lines from White Leghorn origin were established after divergent selection and breeding for high (H line) or low (L line) total (IgM and IgG, but not IgA) antibody titers to KLH in plasma at 16 wk of age (Berghof et al., 2018). These total antibody titers were measured in birds without KLH immunization and are therefore regarded as NAb titers. Non-immunized, ‘normal and healthy’ chickens have antibodies to various exogenous (Parmentier et al., 2004; Cotter et al., 2005), and self-antigens (Jalkanen et al., 1983; Neu et al., 1984; Barua and Yoshimura, 2001; Parmentier et al., 2004, 2017; Bao et al., 2016) including phosphoryl choline (PC; Jalkanen et al., 1983).

Origin, induction, and function of NAb and N(A)Ab are debated. They may operate in innate immunity, but also cooperate with specific components (Baumgarth et al., 2005). Various specific immune responses in mammals (Tomer and Shoenfeld, 1988; Thornton et al., 1994; Ochsenbein et al., 1999; Stäger et al., 2003) and poultry (Lammers et al., 2004) are enhanced by or positively correlated with high levels of NAb and N(A)Ab, and NAb may underlie protective immunity (Zinkernagel, 2012). The NAb and N(A)Ab repertoire and levels may be shaped by continuous polyclonal stimulation by exogenous microbes (Khasbiullina et al., 2018) initiating cross-reactivity-driven responses of auto-reactive B cells, or correspond with the secretion by naturally occurring (auto-reactive) B cell clones, or both (Avrameas et al., 1983; Avrameas, 1991). Higher NAb and N(A)Ab levels in plasma from ‘old’ chickens correspond with the idea that exogenous stimuli enhance the formation of these antibodies (Prokesova et al., 1996), as is true for the enhancement of NAb levels by dietary probiotics (Haghighi et al., 2006). On the other hand, the reported (enhanced) binding of plasma antibodies to self-antigens such as OVA, and the binding of tissue antigens (Parmentier et al., 2017) correspond with the proposed naturally occurring B cell reactivity based on a random gene conversion. The presence of high levels of N(A)Ab in serum and mucosa from mammals and birds, and the high frequency of ‘auto-immune’ idiotypes suggest important effector and regulatory functions of N(A)Ab (Quan et al., 1997). Most N(A)Ab are polyreactive to phylogenetically conserved structures: nucleic acids, heat shock proteins, carbohydrates, phospholipids (Boes, 2000), and (autologous) ABO blood group antigens (Spalter et al., 1999). Apart from binding to evolutionary conserved molecules inside cells—actin, myosin, tubulin, and DNA—N(A)Ab also bind CD5, CD4, CD8, other antibodies, MHC (Berczi et al., 1998), interferon-γ (Caruso and Turano, 1997), and IL-2 (Balsari and Caruso, 1997) arguing for an immune regulatory role for N(A)Ab limiting the intensity and/or duration of immune responses.

Levels of NAb (Berghof et al., 2015) and N(A)Ab (Bao et al., 2016) in poultry are heritable. In addition, a significant non-inherited maternal effect on IgM titers binding both KLH and auto-antigens at 16 wk of age was found, suggesting to be defined maternal component(s), likely present in eggs, which affect antibody titers in progeny. Finally, Western blot analysis on the auto-immune profile in parents and their full sib progeny suggested correspondent reaction patterns of hens and chicks for IgG antibodies but not for IgM (Parmentier et al., 2017).

In the present study, IgM and IgG antibodies were found in adult hen plasma (Table 1), hatchlings (Table 4), and day 8 birds (Table 5) to all tested antigens, but differences with respect to levels and isotypes were found for yolk (Table 2) and albumen (Table 3), which was illustrated by very low titers or even absence of IgG in albumen and IgM in yolk. It is known that IgG is present in yolk, and IgM (and IgA) are absent in yolk (Leslie & Clem, 1969; Rose et al., 1974), whereas IgA and IgM are present in albumen, due to mucosal secretion in the oviduct (Rose et al., 1974). Significant line differences of antibody titers in adult hens were found. Thus, divergent breeding for high or low levels of NAb binding KLH also affected the levels of IgM and IgG antibodies to the other self (OVA), non-self (dsDNA), and endangered (PC-) self-antigens tested.

Table 4 shows that in general, IgM and IgG antibodies to all antigens are present in hatchlings, although considerably lower levels compared to the adult hens. Remarkably, KLH IgM antibodies were absent in the hatchlings from both lines. It is tempting to speculate that the source or origin of IgG antibodies at hatch are thus maternal antibodies transferred via the egg yolk. Our study did not indicate newly formed neonatal antibodies present at hatch, albeit IgG-secreting B cells were reported in chicks from 6 d post-hatch (Lawrence et al., 1981) and neonatal synthesized IgM and IgA antibodies were detected in the plasma of 3- to 4-day-old and 12-day-old chicks, respectively (Leslie and Martin, 1973). IgG appears at the 4th day of embryogenesis and persists till the 16th day (Kramer and Cho, 1970). Also according to Kaspers et al., (1991), the yolk of freshly laid eggs contains only IgG, apart from IgA traces, and maternal transfer of IgG antibodies in chicken mainly happens through yolk or yolk sac (Grindstaff et al., 2003) after which maternal IgG antibodies will be catabolized in the post-hatched chick (Grindstaff et al., 2003; Niewiesk, 2014). Endogenous antibody production starts at 5 d of age, and chickens rely for immune protection on endogenously produced antibodies from 14 d of age onwards (Grindstaff et al., 2003). The low correlations between albumen and hatchling in the present study, however, suggest that the IgM titers in hatchlings may not be of maternal origin. In day 8 chicks, plasma antibody titers (Table 4) are lower compared to those in adult hens (Table 1), and also lower than titers at hatch (Table 3) also suggesting a metabolic breakdown of maternal antibodies and no new detectable production of antibodies yet. Our findings contradict with Hamal et al. (2006), who reported a significant increase from very low levels of IgM antibodies from day 3 to day 7 of age. The significant line differences in all compartments are in line with the selection procedure that aimed for higher antibody production in the high line compared to the low line.

In almost all compartments: yolk (Table 2), albumen (Table 3), hatchlings (Table 4), and day 8 birds (Table 5), high titers of IgG binding OVA were found suggesting that adult hens produce high levels IgG binding OVA that are apparently transferred via the yolk to progeny but are still found in the albumen. This suggests a naturally occurring B cell reactivity by which OVA is recognized as prominent self-antigen, as ovalbumin is already present in the early phase of embryonic development. Antibodies to self-antigens may act as an ‘extraordinary tool for self-assessment’ (Avrameas et al., 1981) that provide tolerance in the adult life (Lacroix-Desmazes et al., 1998), and prevent pathological autoimmunity by binding to epitopes that are similar and even identical with self-antigens (Cohen and Cooke, 1986). In the present study, the IgM titer to PC-OVA (danger-antigen) was higher in the H line compared to the L line against PC-OVA, but this was not true for the ‘not danger-antigen’ OVA (Table 3). Our data suggest that hens also provide chicks with homeostatic antibodies to perform self-assessment.

Similar binding patterns and line differences between adult hen plasma (Figure  1A) and hatchlings (Figure  1B) were detected although not all antigens showed significant line effects. Table 8 shows the correlation of antibody titers for all antigens and isotypes tested between adult hen plasma, yolk, and hatchling plasma. This table suggests that IgG antibodies specific for natural and auto-antigens (KLH, OVA, and PC-OVA) are transferred from hen to yolk and from yolk to hatchling. These correlation values indicate that IgG antibody titers in the hen can predict antibody IgG titers in her offspring via the yolk route. It is tempting to speculate that IgG auto-antibodies might act as an adaptive mechanism to clear intercellular debris (waste) and disease-induced damaged tissue antigens (Nagele et al., 2013). It remains unclear whether IgM antibodies binding dsDNA and PC-OVA are maternal or neonatal of origin, since they are absent in the yolk and low in the albumen. In mice, antigen-immunized mothers induce antigen-reactive IgM antibodies in the offspring (Lemke et al., 2004). In the present study, the albumen route (hen plasma–albumen–hatchling plasma) showed no significant correlated values (Table 9). These results suggest that there is no relation of NAb and N(A)Ab (IgM) levels between hen plasma–albumen–hatchling plasma. IgM (and IgA) were proposed to end up in the residual yolk of hatchlings (Kaspers et al., 1996), which was not studied at present.

Titers of IgM NAb binding KLH are heritable in poultry (Berghof et al., 2015) and affected by a non-genetic maternal environmental effect. In the present study, KLH IgM-titers in plasma of hatchlings and of day 8 birds were absent, but IgG titers to KLH were found in plasma at hatch and in day 8 birds, albeit significantly higher in the H line. Thus, the maternal environmental effect on IgM binding KLH titers in the selection lines (Berghof et al., 2015) is therefore likely not based on transfer of maternal IgM antibodies via the egg (neither yolk nor albumen route), and production of neonatal IgM antibodies likely starts after the first week of life in an accelerated fashion in H-line chicks. In mice, the mother epigenetically transfers its antibody repertoire to offspring based on the mother's immunological knowledge of her antigenic environment (Lemke et al., 2009) likely via IgG. The role of maternal IgG binding KLH as the epigenetic factor affecting IgM antibodies binding KLH in chickens thus remains to be studied. Hens transfer IgG antibodies into their eggs in proportion to the concentration in their circulation (Blount et al., 2002; Grindstaff et al., 2003; Grindstaff, 2008). This proportion has been related to the repeated exposures to specific antigens: the higher the exposure of the antigens, the higher concentration of antibodies that will be likely transported to the offspring (Hasselquist and Nilsson, 2009). The current data suggest that genetic background also affects the levels of maternal antibodies transferred via the egg.

Table 9 shows the comparison of the composite trade PC-BSA IgM/dsDNA IgG in each compartment. High levels of IgG anti-dsDNA antibodies may downregulate the production of IgM anti-PC antibodies suggesting a biomarker for health status (López et al., 2016). In man, elevated anti-dsDNA IgG antibodies are the hallmark of systemic lupus erythematosus (**SLE**) disease (Pisetsky, 2008; Grönwall et al., 2012; López et al., 2016) and renal problems (Pisetsky, 2008). In the present study, the highest value of this composite trait was found in adult hens, compared to the other compartments. No significant line difference was found. Therefore, this composite trait does not reflect immune status and a different health status.

In conclusion, we verified the presence of IgM and IgG antibodies binding (dangerous) self- and foreign antigens such as PC-BSA, KLH, dsDNA, PC-OVA, and OVA in plasma from adult hens, yolk (IgG only), albumen (IgM only), plasma from hatchlings, and plasma from day 8 chicks. The NAb line selection affected antibody titers in each compartment. Maternal IgG transfer is suggested for specific antigens and this isotype provides the young chick passively with immunoglobulins (NAb and N(A)Ab) that were proposed to perform a variety of homeostatic functions. It has to be kept in mind, however, that this study is based on divergently selected but not ad random specimens and a limited set of antigens tested. Western blot analysis is underway to distinguish trans-epigenetically transferred maternal N(A)Ab from newly formed neonatal N(A)Ab. A composite trait of IgM PC-BSA and IgG dsDNA could as yet not be related with immune status of the lines studied.

## APPENDIX

**Appendix 1.** Coating concentrations of antigens and conjugate dilutions used.

		Antigen Coating	Serum Starting	Dilution		Conjugate
Antigen	Sample	Concentration (μg/mL)	Dilution	Steps	Conjugate	Dilution
PC-BSA	Hen Plasma	1	40	4	IgM	10,000
			40	4	IgG	10,000
	Yolk	2	10	2	IgM	10,000
			10	2	IgG	10,000
	Albumen	2	10	2	IgM	10,000
			10	2	IgG	10,000
	Hatchling Plasma	1	40	4	IgM	10,000
			40	4	IgG	10,000
	Day 8 Plasma	1	40	4	IgM	5,000
			40	4	IgG	20,000
KLH	Hen Plasma	1	40	4	IgM	10,000
			40	4	IgG	10,000
	Yolk	1	40	4	IgM	10,000
			40	4	IgG	10,000
	Albumen	1	10	2	IgM	10,000
			10	2	IgG	10,000
	Hatchling Plasma	1	40	4	IgM	10,000
			40	4	IgG	10,000
	Day 8 Plasma	1	40	4	IgM	5,000
			40	4	IgG	10,000
dsDNA	Hen Plasma	0.5	40	4	IgM	10,000
			40	4	IgG	10,000
	Yolk	2	10	2	IgM	5,000
			10	2	IgG	5,000
	Albumen	2	10	2	IgM	5,000
			10	2	IgG	5,000
	Hatchling Plasma	0.5	40	4	IgM	10,000
			40	4	IgG	10,000
	Day 8 Plasma	1	20	2	IgM	5,000
			20	2	IgG	5,000
PC-OVA	Hen Plasma	1	40	4	IgM	20,000
			40	4	IgG	20,000
	Yolk	1	40	4	IgM	5,000
			40	4	IgG	5,000
	Albumen	1	10	2	IgM	5,000
			10	2	IgG	5,000
	Hatchling Plasma	1	40	4	IgM	20,000
			40	4	IgG	20,000
	Day 8 Plasma	1	40	4	IgM	5,000
			40	4	IgG	10,000
OVA	Hen Plasma	1	40	4	IgM	20,000
			40	4	IgG	20,000
	Yolk	1	40	4	IgM	10,000
			40	4	IgG	10,000
	Albumen	1	10	2	IgM	10,000
			10	2	IgG	10,000
	Hatchling Plasma	1	40	4	IgM	20,000
			40	4	IgG	20,000
	Day 8 Plasma	1	20	2	IgM	5,000
			10	2	IgG	10,000
